# Gold Nanoparticle-Aptamer-Based LSPR Sensing of Ochratoxin A at a Widened Detection Range by Double Calibration Curve Method

**DOI:** 10.3389/fchem.2018.00094

**Published:** 2018-04-04

**Authors:** Boshi Liu, Renliang Huang, Yanjun Yu, Rongxin Su, Wei Qi, Zhimin He

**Affiliations:** ^1^State Key Laboratory of Chemical Engineering, School of Chemical Engineering and Technology, Tianjin University, Tianjin, China; ^2^School of Environmental Science and Engineering, Tianjin University, Tianjin, China; ^3^Collaborative Innovation Center of Chemical Science and Engineering, Tianjin, China

**Keywords:** aptamer, LSPR, ochratoxin A, AuNP aggregation, colorimetric detection

## Abstract

Ochratoxin A (OTA) is a type of mycotoxin generated from the metabolism of *Aspergillus* and *Penicillium*, and is extremely toxic to humans, livestock, and poultry. However, traditional assays for the detection of OTA are expensive and complicated. Other than OTA aptamer, OTA itself at high concentration can also adsorb on the surface of gold nanoparticles (AuNPs), and further inhibit AuNPs salt aggregation. We herein report a new OTA assay by applying the localized surface plasmon resonance effect of AuNPs and their aggregates. The result obtained from only one single linear calibration curve is not reliable, and so we developed a “double calibration curve” method to address this issue and widen the OTA detection range. A number of other analytes were also examined, and the structural properties of analytes that bind with the AuNPs were further discussed. We found that various considerations must be taken into account in the detection of these analytes when applying AuNP aggregation-based methods due to their different binding strengths.

## Introduction

The ochratoxins constitute a group of structurally similar mycotoxins that are produced mainly by the metabolism of *Aspergillus* and *Penicillium* (Kuiper-Goodman and Scott, 1989; Malir et al., 2016). Within this group, ochratoxin A (OTA), which was discovered, isolated, and identified in 1965, is considered one the most harmful and damaging toxins (van der Merwe et al., 1965; Ha, 2015; Koszegi and Poór, 2016; Malir et al., 2016). More specifically, it exhibits nephrotoxic, hepatotoxic, embryotoxic, teratogenic, neurotoxic, immunotoxic, genotoxic, and carcinogenic effects in humans, livestock, and poultry (Kuiper-Goodman and Scott, 1989; IARC, 1993; O'Brien and Dietrich, 2005; Binder et al., 2007). In addition, OTA was classified as a class 2B carcinogen in 1993 by the International Agency for Research on Cancer (IARC, 1993), and many countries have set a maximum limit for OTA contents in food. For example, the maximum permitted OTA content in cereals, beans, and their products is 5.0 μg/kg, as specified by the Ministry of Health of the People's Republic of China (Ministry of Health, 2011). However, as the OTA-producing microorganisms are ubiquitous in nature and OTA itself is difficult to degrade, OTA can contaminate a range of agricultural products, including grains, beans, nuts, and wheat (O'Brien and Dietrich, 2005; Binder et al., 2007; Santos et al., 2009; Koszegi and Poór, 2016), and so the development of a rapid and simple OTA detection method is of particular importance in the context of food safety.

Since the first report into the quantitative detection of OTA was published in 1973 (Nesheim et al., 1973), a number of additional OTA detection methods have been developed based on thin-layer chromatography (Nesheim et al., 1973), high performance liquid chromatography (HPLC) (Molinié et al., 2005), gas chromatography (Jiao et al., 1992), and mass spectrometry (Ediage et al., 2012; Cramer et al., 2015). Although these methods offer satisfactory sensitivity and selectivity, they tend to require expensive instrumentation, complicated operations, and highly-trained operators. To address these issues, novel OTA detection methods have been developed in recent years, including enzyme-linked immunosorbent assays (Liu et al., 2008), surface plasmon resonance methods (Zhu et al., 2015), polymerase chain reaction (Sanzani et al., 2015), fluorescent method (Yao et al., 2015), electrochemiluminescence method (Wang et al., 2016b), and upconversion nanoparticle method (Dai et al., 2017), etc. However, the cost of detection remains high for these methods.

As such, colorimetric detection has recently attracted growing attention because of its simplicity, easy operation, and low cost (Song et al., 2011; Li et al., 2017). Indeed, colorimetric methods based on changes in the localized surface plasmon resonance (LSPR) signal caused by the aggregation of noble metal nanoparticles (NPs) are sensitive, and the detection results can generally be observed by the naked eye (Sepulveda et al., 2009; Mayer and Hafner, 2011; Wang et al., 2011; Zhang et al., 2012, 2014; Chen et al., 2013). In this system, noble metal NPs can be dispersed as a colloid via surface charge repulsion (Saha et al., 2012), but will aggregate in the presence of an electrolyte due to destruction of the charge repulsion. This aggregation alters the LSPR effect, resulting in a red shift of the UV-vis adsorption spectrum.

Aptamers are oligonucleotides screened from nucleic acid libraries, and they are known to exhibit high affinities and selectivities (Hermann and Patel, 2000; Willner and Zayats, 2007; Famulok and Mayer, 2011; Fiore et al., 2015; Pfeiffer and Mayer, 2016; Ruscito and DeRosa, 2016). Compared to traditional antibody recognition elements, aptamers have a number of advantages, such as facile synthesis, low cost, stable properties, and facile chemical modification (Wang et al., 2010; Iliuk et al., 2011), which render them of particular interest in the field of biosensors. At the same time, the base groups of nucleic acids can bind with gold (Demers et al., 2002), and as such, can stabilize gold nanoparticles (AuNPs) to avoid salt aggregation. There are some previously reported OTA aptasensors (Yang et al., 2011; Luan et al., 2015) based on this principle, however, analytes such as OTA can also adsorb on the AuNPs to inhibit salt aggregation. Consequently, the results obtained from only one single linear calibration curve is not reliable. In order to address this issue and widen the OTA detection range, we developed a “double calibration curve” method. Indeed, this system appears particularly innovative compared with previously reported OTA aptasensors (Yang et al., 2011; Luan et al., 2015). In addition, to obtain reliable results and widen the OTA detection range, a “double calibration curve” method will be examined to ultimately produce a label-free, rapid, and cost-competitive method.

## Materials and methods

### Materials

Gold(III) chloride trihydrate (HAuCl_4_·3H_2_O), ochratoxin A (OTA), ochratoxin B (OTB), aflatoxin B1 (AFT B1), and aflatoxin B2 (AFT B2) were purchased from Sigma-Aldrich. Sodium citrate dehydrate, adenosine triphosphate (ATP), 17β-estradiol (EST), and oxytetracycline hydrochloride (OTC·HCl) were purchased from Heowns Biochemical Technology Co., Ltd. The nucleotide sequence of the OTA aptamer was GATCGGGTGTGGGTGGCGTAAAGGGAGCATCGGACA (5′ to 3′) (Cruz-Aguado and Penner, 2008). The sequences of oligonucleotide aptamers for the other analytes are shown in Table S1. These aptamers were synthesized then purified by HPLC (Sangon Biotech, Shanghai, China). Stock solutions (100 μM) of the prepared aptamers were dissolved in ultrapure water then stored at −18°C. All reagents employed were of analytical grade, and all solutions were prepared using ultrapure water (18.2 MΩ·cm^−1^, Sartorius Arium Pro VF, Germany).

### Preparation of the AuNP colloid

A stock solution of HAuCl_4_·3H_2_O (0.34 mL, 10%, w/w) was added to ultrapure water (100 mL) in an Erlenmeyer flask. The resulting mixture was stirred using a magnetic stirrer (>300 rpm) and heated from ambient temperature to (98 ± 1)°C using a water bath. Subsequently, a solution of sodium citrate (2 mL, 194 mM) was added to the flask with continued stirring, which resulted in a gradual change in the solution color from yellow to gray, and finally to dark red. The reaction was stopped 20 min after the addition of sodium citrate, and the resulting AuNP colloid was stored in the absence of light at 4°C.

### Characterization of AuNPs and their aggregates

The morphologies of the AuNPs and their aggregates were characterized using transmission electron microscopy (TEM, JEOL JEM-2100F, Japan), while the particle sizes were determined using a zetasizer (Malvern Nano ZS, UK).

### OTA detection procedure

UV-vis absorbance measurements were performed using a Persee TU-1810 spectrophotometer (Beijing, China). A mixture of the AuNP colloid (200 μL), the analyte (50 μL), and the OTA aptamer solution (1 μM) was mixed thoroughly and incubated for 10 min (all times stated were strictly controlled). After this time, a 0.5 M NaCl solution was added (for details regarding the volumes of OTA aptamer solution and NaCl solution employed, please see the “Optimization of the OTA detection conditions” subsection of the Results and Discussion section) and the solution mixed well. After incubation for a further 3 min, the resulting solution was transferred into a 1 mm cuvette to measure the UV-vis absorbance (450–710 nm). A calibration curve was then plotted using the ratio of absorbances at 630 nm and 520 nm (i.e., A_630_/A_520_) as the ordinate and the OTA concentration as the abscissa. The detection of ATP, EST, and OTC·HCl was carried out as described for the detection of OTA.

## Results and discussion

### Principle of the colorimetric OTA aptasensing method

**Figure 1:**
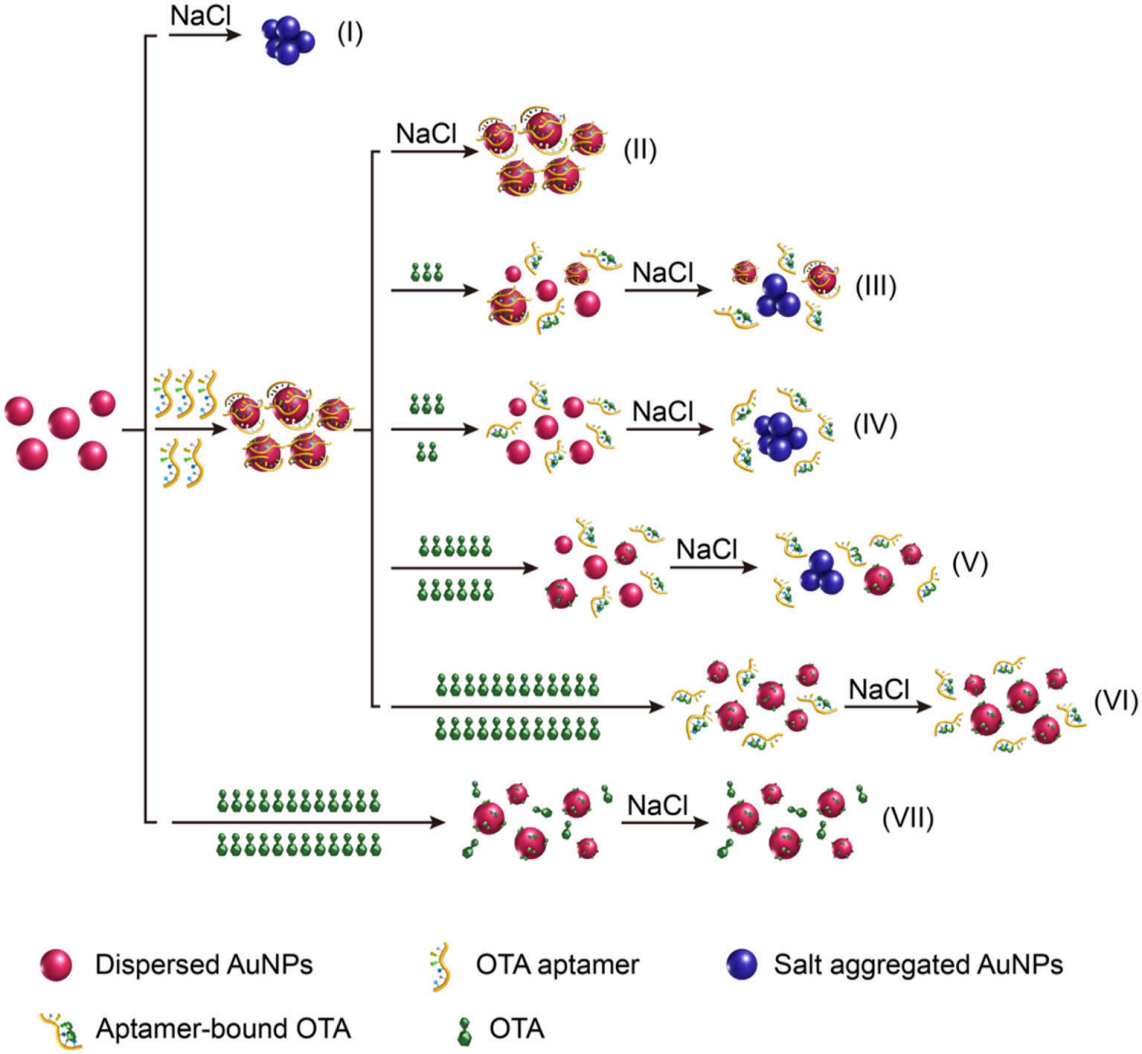
Schematic illustration of the “double calibration curve” colorimetric OTA aptasensing method based on the LSPR effect of AuNPs and their aggregates.

An overview of the LSPR-based colorimetric OTA aptasensing method is illustrated in Figure 1. In this system, the AuNPs prepared from the reaction of HAuCl_4_ with sodium citrate can be stably dispersed in pure water due to the charge repulsion from the surface carboxyl groups, which leads to the formation of a dark red colloidal liquid with a UV-vis wavelength absorbance maximum at ~520 nm. In the presence of electrolytes (such as NaCl) at the appropriate concentration, this charge repulsion can be destroyed, resulting in aggregation of the AuNPs (i.e., salt aggregation). This aggregation causes an increase in absorbance at 630 nm and a change in the AuNP colloid color from dark red to blue/purple (Figure 1I).

As DNA can adsorb on the AuNP surfaces through interactions of their base groups with gold (Demers et al., 2002), which is even stronger than DNA hybridization (Demers et al., 2002; Kimura-Suda et al., 2003; Sponer et al., 2004), DNA molecules can protect the AuNPs from salt aggregation (Liu and Liu, 2017). It was therefore expected that addition of the OTA aptamer would prevent salt aggregation of the AuNP colloids (Figure 1II). However, in the presence of OTA, the OTA aptamer will dissociate from the AuNPs as the aptamer is folded, so that its binding to AuNPs is kinetically hindered. Thus, upon the addition of NaCl, the AuNPs will aggregate due to the loss of protection, thereby resulting in a color change to blue/purple, and an increase in absorbance at 630 nm (Figures 1III, IV). Indeed, under specific conditions, A_630_/A_520_ exhibits a linear relationship with the OTA concentration, thereby allowing a calibration curve to be obtained for OTA detection.

Interestingly, we found that upon increasing the OTA concentration, the A_630_/A_520_ ratio reached a maximum prior to decreasing once again, thereby indicating that the aggregation level decreased after reaching a maximum. To the best of our knowledge, this constitutes the first report of this phenomenon to date. Indeed, we expect that this occurs due to protection of the AuNPs from salt aggregation by the presence of OTA (Figures 1V, VI). Furthermore, in the presence of high OTA concentrations but in the absence of the OTA aptamer, the A_630_/A_520_ value was low, which indicated a low level of aggregation, thereby confirming that salt aggregation can be successfully inhibited by OTA (Figure 1VII).

### Optimization of the OTA detection conditions

The detection of OTA was based on the equilibrium between NaCl-induced AuNP aggregation and the inhibition of aggregation by the OTA aptamer. Therefore, determination of the optimal detection conditions included optimization of both the NaCl and OTA aptamer contents. In the absence of the OTA aptamer, the A_630_/A_520_ ratio increased significantly upon increasing the quantity of 0.5 M NaCl solution prior to reaching a plateau (Figure S1A). As indicated, the optimal volume of the 0.5 M NaCl solution for OTA detection was 17 μL.

Subsequently, the influence of the 1 μM OTA aptamer solution volume was examined, and it was found that the A_630_/A_520_ ratio initially decreased upon increasing the volume of aptamer solution employed, with a plateau being reached at volumes >10 μL. Based on these results, an optimal aptamer solution volume of 10 μL was selected (Figure S1B).

### Calibration curve for OTA detection at low concentrations

**Figure 2:**
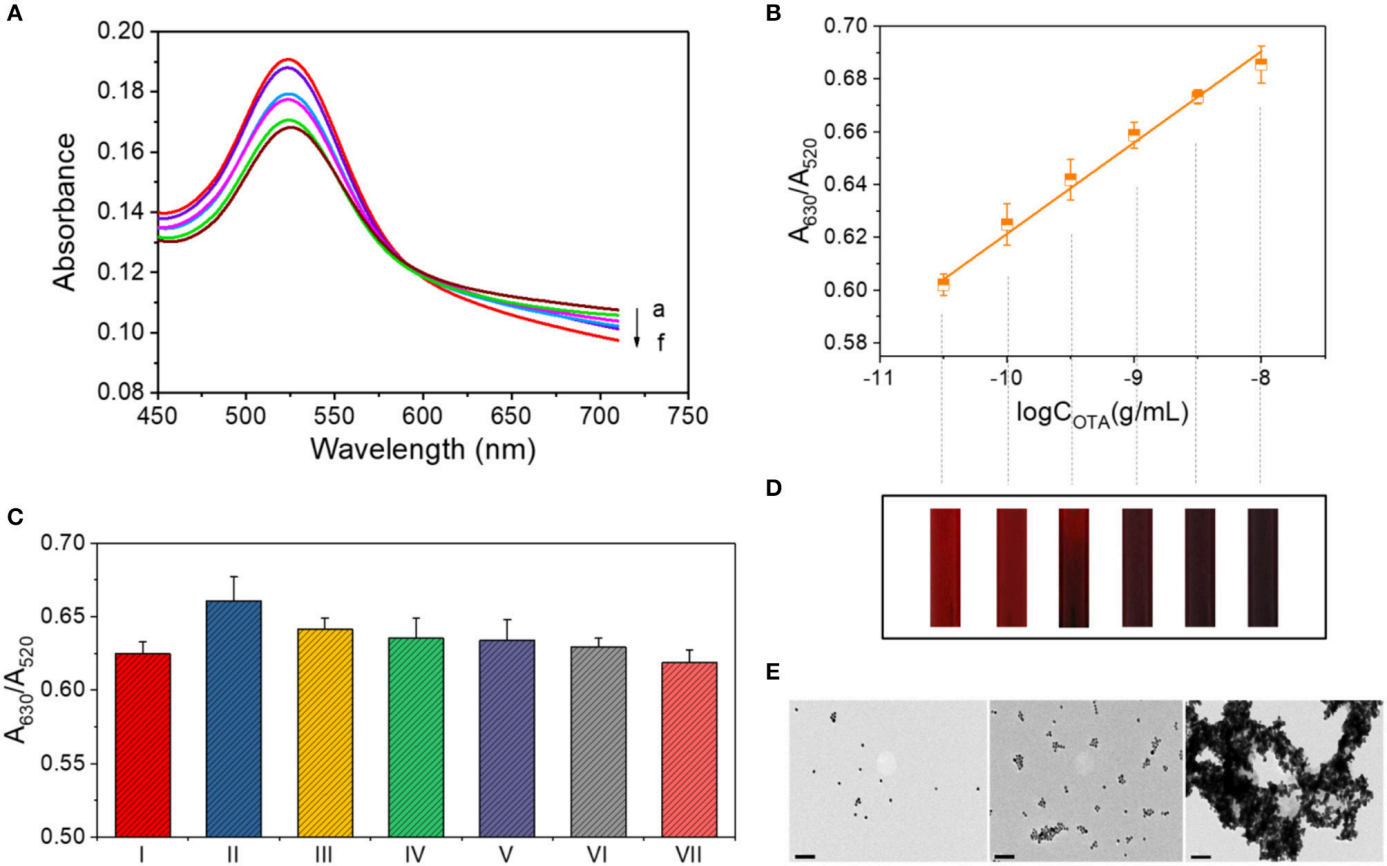
OTA assay for concentrations ranging from 10^−10.5^ to 10^−8^ g/mL. **(A)** UV-vis spectrum for OTA detection. Absorbance curves (a–f) correspond to samples containing 10^−8^, 10^−8.5^, 10^−9^, 10^−9.5^, 10^−10^, and 10^−10.5^ g/mL OTA, respectively. **(B)** Calibration curve for OTA detection at concentrations between 10^−10.5^ and 10^−8^ g/mL. **(C)** Selectivity of the OTA assay in the low concentration range. The various columns correspond to the following detection systems: I: 0.1 ng/mL OTA. II: 0.1 ng/mL OTA + 1 ng/mL OTB. III: 0.1 ng/mL OTA + 1 ng/mL AFT A. IV: 0.1 ng/mL OTA + 1 ng/mL AFT B. V: 0.1 ng/mL OTA + 1 ng/mL OTC·HCl. VI: 0.1 ng/mL OTA + 1 mg/mL maltose. VII: 0.1 ng/mL OTA + 1 mg/mL sucrose. **(D)** The color of each solution corresponding to the data points of part B. **(E)** TEM images of the AuNP aggregates in the OTA detection systems at concentrations of 0, 10^−10^, and 10^−8^ g/mL (from left to right). Scale bar = 100 nm.

The absorbance curves for OTA detection were obtained under the optimized conditions as shown in Figure 2A. Upon increasing the OTA concentration, A_630_ increased, while A_520_ decreased, thereby resulting in a linear relationship (y = 0.0346x + 0.9673, *R*^2^ = 0.993) between the A_630_/A_520_ ratio and the logarithm of the OTA concentration (Figure 2B). As use of the standard deviation is not appropriate for evaluating the limit of detection (LOD) of a colorimetric quantitative detection method (LOD = 3 × the standard deviation of blank sample / the slope of the calibration curve), we herein calculated the LOD as the lowest value of the linear range (i.e., 10^−10.5^ g/mL). To verify the LOD, we performmed the control experiment using the complimentary single-stranded DNA of the OTA aptamer. The contrast of A_630_/A_520_ values is shown in Figure S2. In addition, the color change taking place during detection is shown in Figure 2D, where each band corresponds to a data point from Figure 2B. The theory behind this system is similar to that described in previous studies by Yang et al. (2011) and Luan et al. (2015), although some differences exist in the details.

In addition, the morphologies of the AuNPs and their aggregates in the detection systems containing 0, 10^−10^, and 10^−8^ g/mL OTA were then characterized by TEM. As shown in Figure 2E, the AuNPs were well dispersed in the absence of OTA, while low-level aggregation occurred in the presence of 10^−10^ g/mL OTA, and significant aggregation occurred upon the addition of 10^−8^ g/mL OTA.

### Validation of the selectivity

We subsequently compared the signals observed for the samples containing 0.1 ng/mL OTA and a series of interfering substances, including 1 ng/mL OTB, 1 ng/mL AFT, 1 ng/mL AFT B, 1 ng/mL OTC·HCl, 1 mg/mL maltose, and 1 mg/mL sucrose (Figure 2C). As indicated, the addition of other interfering substances had no significant effect on the signal. There was a special case for OTB, which is structurally similar to OTA with only difference in the absence of a chlorine group on the benzopyran ring (Figure S3), affected the response signal of this detection method.

### Double calibration curves for the OTA assay method

**Figure 3:**
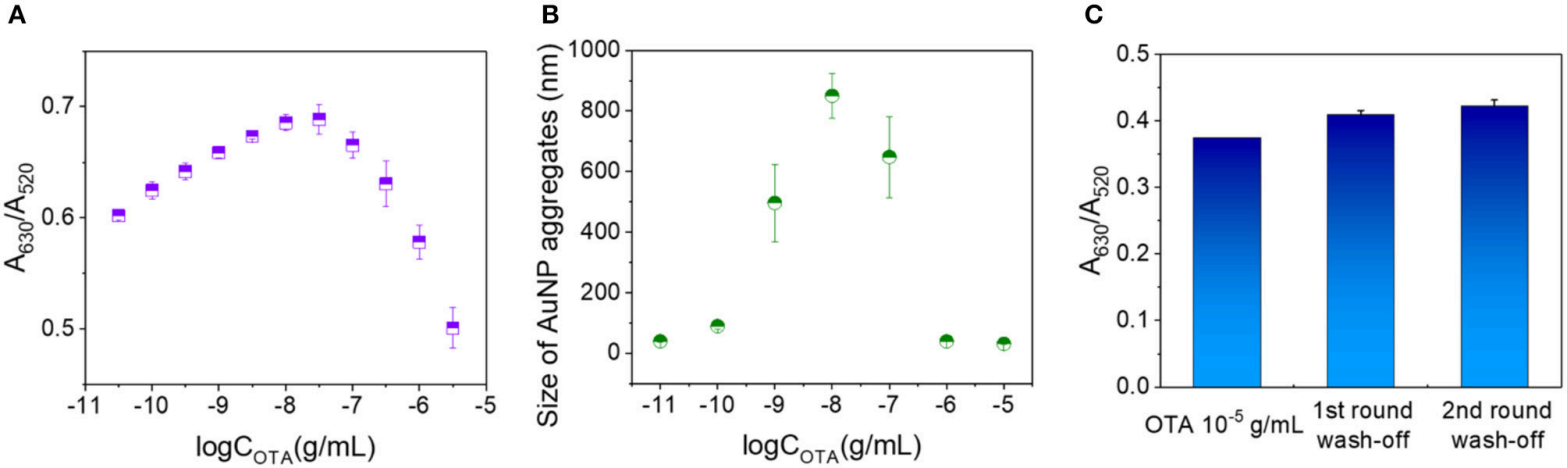
Examination of the decrease in A_630_/A_520_ with OTA concentration above a critical value. **(A)** A_630_/A_520_ values corresponding to OTA concentrations ranging from 10^−11^ to 10^−5.5^ g/mL. **(B)** Particle sizes of the AuNP aggregates at OTA concentrations ranging from 10^−11^ to 10^−5^ g/mL. **(C)** A_630_/A_520_ values of the 10^−5^ g/mL OTA detection systems prior to washing, and after washing once and twice with water.

Under the optimized conditions described above, the concentration of OTA in the analytes was further increased gradually to 10^−5^ g/mL. As shown in Figure 3A, the A_630_/A_520_ ratio rose up to an OTA concentration of 10^−7.5^ g/mL prior to decreasing at higher concentrations. The average sizes of the AuNP aggregates formed in the presence of different OTA concentrations were then measured based on the dynamic scattering effect. Interestingly, the size of the AuNP aggregates followed a similar trend to the change in A_630_/A_520_ (i.e., an initial increase followed by a subsequent decrease), with the highest degree of aggregation being observed at an OTA concentration of ~10^−8^ g/mL (Figure 3B). These results indicate that at low OTA concentrations, a number of OTA aptamers remained adsorbed on the AuNPs, thereby protecting them from salt aggregation. However, upon increasing the OTA concentration, the aptamers gradually became saturated by the OTA, thereby resulting in their dissociation from the AuNPs, which promoted salt aggregation. Upon further increasing the OTA concentration, a degree of free OTA remained in the system, and it was expected that this resulted in some stabilization of the AuNPs against salt aggregation, thereby accounting for the reduction in particle size.

To investigate the potential stabilization of AuNPs by OTA, a washing stage was introduced (see Data Sheet 1 in the Supporting Information for experimental details), and the obtained A_630_/A_520_ ratios after each washing are shown in Figure 3C. More specifically, prior to washing, the A_630_/A_520_ value was 0.374, and this value increased to 0.409 and 0.422 after washing (A_630_/A_520_ = 0.966 in the absence of OTA), thereby suggesting that washing did not remove significant quantities of OTA from the detection system. These results therefore indicate that OTA binds to the AuNP surfaces via interactions that cannot be destroyed by water washing, and so salt aggregation of the AuNPs is prevented in the presence of OTA.

**Figure 4:**
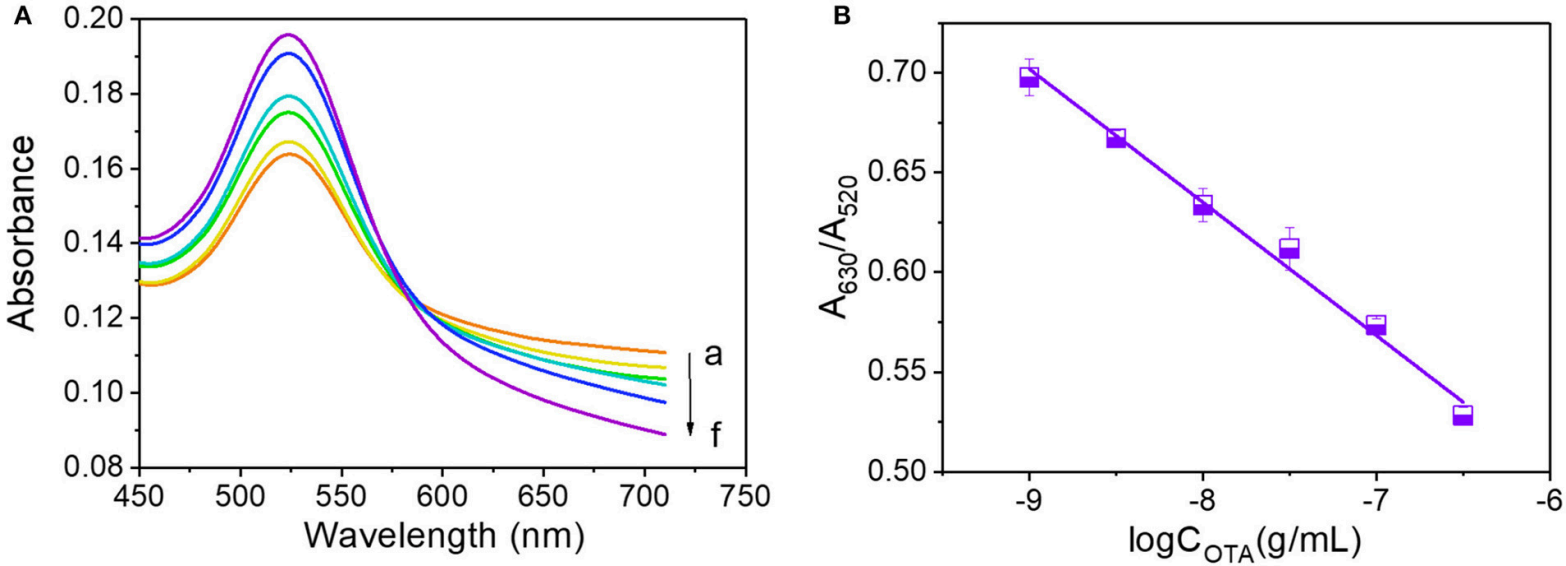
OTA analysis at concentrations ranging from 10^−9^ to 10^−6.5^ g/mL. **(A)** UV-vis spectra of OTA detection. Absorbance curves (a–g) correspond to OTA concentrations of 10^−9^, 10^−8.5^, 10^−8^, 10^−7.5^, 10^−7^, and 10^−6.5^ g/mL, respectively. **(B)** Calibration curve for OTA detection at concentrations ranging from 10^−9^ to 10^−6.5^ g/mL.

Due to the observed reduction in A_630_/A_520_ with an increase in OTA concentration above the critical value, we investigated the effects of high OTA concentrations by reducing the volumes of the 0.5 M NaCl and OTA aptamer solutions to 14 and 5 μL, respectively. The resulting UV-vis absorbance spectra observed between OTA concentrations of 10^−9^ and 10^−6.5^ g/mL are shown in Figure 4A, and a calibration curve to demonstrate the relationship between A_630_/A_520_ and the OTA concentration was plotted (Figure 4B). In this case, the corresponding equation for the linear relationship was y = −0.0668x + 0.1009, and the *R*^2^ value was 0.990.

**Figure 5:**
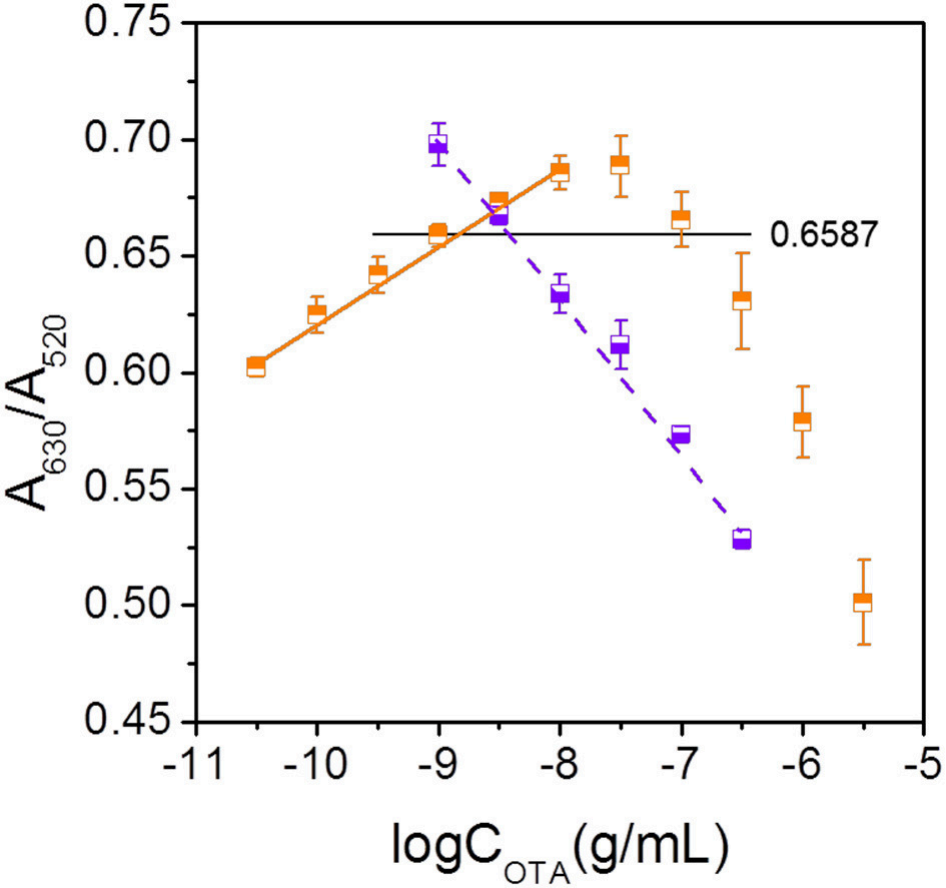
“Double calibration curve” method showing the calibration curves obtained at low (solid line) and high (dashed line) OTA concentrations.

As indicated in Figures 3A, 5, the increase in the OTA concentration resulted in an initial increase of A_630_/A_520_, which was followed by a later decrease. As such, this single linear calibration curve method is not suitable for the calculation of detected OTA concentrations when applying the method based on changes in the AuNP LSPR signal change caused by salt aggregation. Thus, we proposed a “double calibration curve” method to address this issue, as outlined in Figure 5. More specifically, the OTA-containing analyte was initially assayed under the conditions corresponding to the solid calibration curve, and the A_630_/A_520_ value is defined as x_1_. When x_1_ > 0.6587 (0.6587 is the A_630_/A_520_ value corresponding to a concentration of 10^−9^ g/mL on the solid calibration curve), the OTA concentration in the analyte must be higher than 10^−9^ g/mL, so the dashed calibration curve and its assay conditions are suitable for OTA detection. When x_1_ < 0.6587, a ten-fold dilution is applied to the analyte, and the A_630_/A_520_ is defined as x_2_. When x_2_ <x _1_, which indicated that the original concentration of OTA in the analyte is lower than 10^−9^ g/mL, the solid calibration curve and its assay conditions can be applied in the detection of OTA. And when x_2_ > x_1_, the concentration of OTA in the original analyte must be higher than 10^−8^ g/mL, so the dashed calibration curve should be applied for OTA detection. Thus, reliable results can be obtained by combination of these two calibration curves, and using this system, the concentration range could be widened from 10^−10.5^-10^−8^ to 10^−10.5^-10^−6.5^ g/mL. The detection range is wider than the ranges of some reported methods (Table 1). The additional analysis time required for this “double calibration curve” method was <15 min.

**Table 1:** Comparison of some OTA aptasensors in detection range.

**Method**	**Detection range**	**Reference**
Colorimetric	0.05–50 ng/mL	Luan et al., 2015
Surface plasmon resonance	0–10 ng/mL	Zhu et al., 2015
Colorimetric	0.5–100 ng/mL	Wang et al., 2016a
Colorimetric	0.0316–316 ng/mL	Our work

To validate the application of this “double calibration curve” method, corn was selected in a real sample analysis. In this experiment, firstly, 10 g corn was added to 500 mL water, and fully grinded to get a mixture. Then OTA was added to two portions of the above mixture to make the final concentration as 0.1 and 10 ng/mL, respectively. Each spiked sample was filtered through a membrane with a pore diameter of 0.22 μm, and then ultrafiltered through 3500 Dalton membrane. The results got via “double calibration curve” method are shown in Table 2. These results indicated that the “double calibration curve” method can offer effective and reliable analysis of OTA.

**Table 2:** Detection results of OTA in corn samples using “Double calibration curve” method (*n* = 3).

**Sample**	**Spiked (ng/mL)**	**Total detected (ng/mL)**	**Recovery (%)**	**RSD (%)**
**1**	0.1	0.110	110.0	10.19
**2**	10	9.927	99.3	14.01

**Figure 6:**
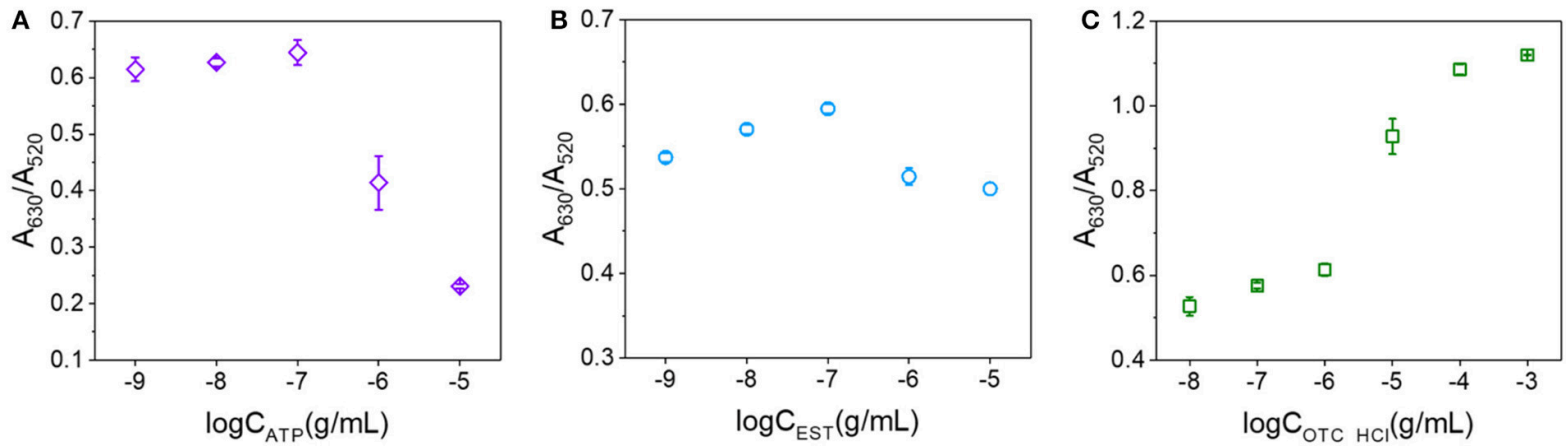
Relationships between the A_630_/A_520_ ratios and analyte concentrations. **(A)** For ATP concentrations ranging from 10^−9^ to 10^−5^ g/mL. **(B)** For EST concentrations ranging from 10^−9^ to 10^−5^ g/mL. **(C)** For OTC·HCl concentrations ranging from 10^−8^ to 10^−3^ g/mL.

In addition to OTA, we expected that a number of other analytes may also be capable of binding to the surfaces of AuNPs and preventing salt aggregation. Thus, we herein examined ATP, EST, and OTC·HCl using their corresponding aptamers (shown in Table S1) and the conditions corresponding to the solid line in Figure 5. The relationships between the A_630_/A_520_ ratios and the analyte concentrations are shown in Figure 6. As indicated, ATP exhibited a similar trend to OTA in terms of preventing the salt aggregation of AuNPs. However, EST exhibited only a weak effect, while in contrast, OTC·HCl strengthened the salt aggregation effect. Examination of the structures of the various analytes (see Figure S3) was then carried out to obtain some insight into the different behaviors of these compounds. In this context, we note that Demers et al. reported that the base groups of DNA have an affinity to gold (Demers et al., 2002) which is stronger than the hybridization of DNA (Sponer et al., 2004), thereby allowing their adsorption on the surfaces of AuNPs. In addition, a series of studies by the Kumar group (Kumar et al., 2002; Ramanath et al., 2004; Maddanimath et al., 2005) indicated that interactions can also take place between gold and aromatic rings. It therefore appeared that the enhanced affinity of OTA toward the AuNPs compared to EST and OTC was likely due to the presence of two aromatic rings in the OTA molecule, compared to one in each EST and OTC molecule. In addition, in both EST and OTC, the aromatic ring takes the form of a heterocyclic ring, while in OTA, a phenyl (i.e., carbon only) ring exists in addition to a heterocyclic ring, and the affinity of this phenyl group to gold may be stronger than that of the heterocyclic rings due to stronger conjugated π bond. Thus, upon application of the colorimetric aptasensing method in the detection of various analytes, it must first be considered whether the target molecule can bind with the AuNPs to prevent salt aggregation and ultimately ensure an accurate and quantitative assay.

## Conclusions

We herein reported the development of a novel assay method for the mycotoxin ochratoxin A (OTA) based on the localized surface plasmon resonance (LSPR) effect of gold nanoparticles (AuNPs) and their aggregates. We found that both OTA and its aptamer could bind to the AuNPs to inhibit salt (i.e., electrolyte-induced) aggregation of the NPs. As such, the use of a single linear calibration curve was not suitable for calculation the OTA concentration in a sample, and so we proposed and developed a “double calibration curve” method to address this issue, and to expand the detection range. We also examined the detection of a number of other analytes and found that in addition to OTA, adenosine triphosphate (ATP), and 17β-estradiol (EST) can also bind with AuNPs through the affinity of gold to nucleotide base groups and aromatic rings. However, due to their varying binding strengths, various considerations must be taken into account when applying this LSPR-based colorimetric aptasensing method to the assay of analytes that can prevent or promote nanoparticle salt aggregation. These results are of particular importance as traditional assays for the detection of OTA are expensive and complicated, and we propose that our rapid and low-cost method will be applicable in the detection of this mycotoxin in foods samples for humans, livestock, and poultry.

## Author contributions

BL, RH, and RS designed research; BL performed research; all the authors analyzed data and wrote the paper. All the authors read and approved the final manuscript.

### Conflict of interest statement

The authors declare that the research was conducted in the absence of any commercial or financial relationships that could be construed as a potential conflict of interest.
