# Supplementary material for: Gold Nanoparticle-Aptamer-Based LSPR Sensing of Ochratoxin A at a Widened Detection Range by Double Calibration Curve Method
Source: Front Chem. 2018 Apr 4;6:94. doi: 10.3389/fchem.2018.00094 (PMC5893832; doi:10.3389/fchem.2018.00094)
Supplement: Supplementary file 1 [file Image1.PDF]

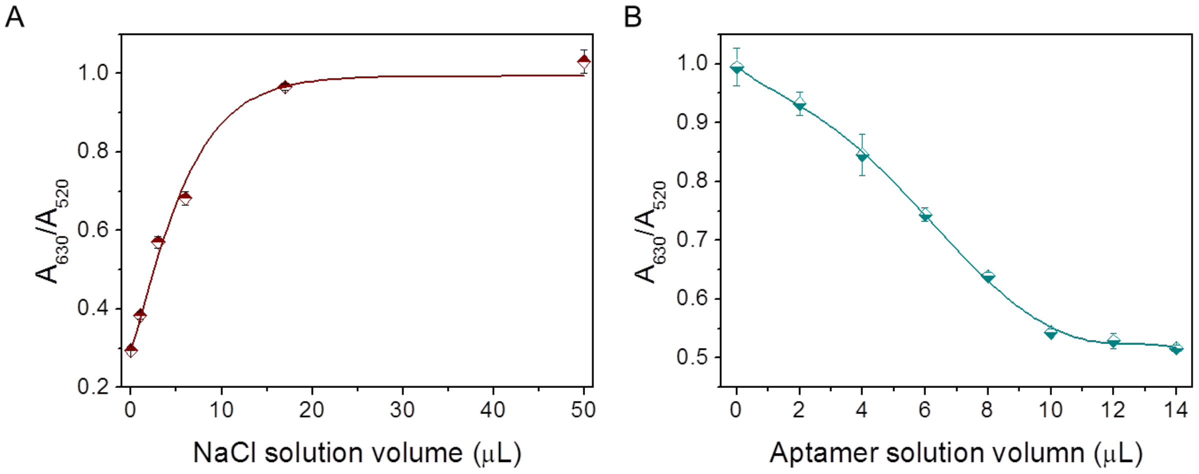

Figure S1: Optimization of the OTA detection conditions. A: Optimization of the volume of the 0.5 M NaCl solution; B: Optimization of the volume of the 1 μM OTA aptamer solution.
