# Supplementary material for: Gold Nanoparticle-Aptamer-Based LSPR Sensing of Ochratoxin A at a Widened Detection Range by Double Calibration Curve Method
Source: Front Chem. 2018 Apr 4;6:94. doi: 10.3389/fchem.2018.00094 (PMC5893832; doi:10.3389/fchem.2018.00094)
Supplement: Supplementary file 2 [file Image2.PDF]

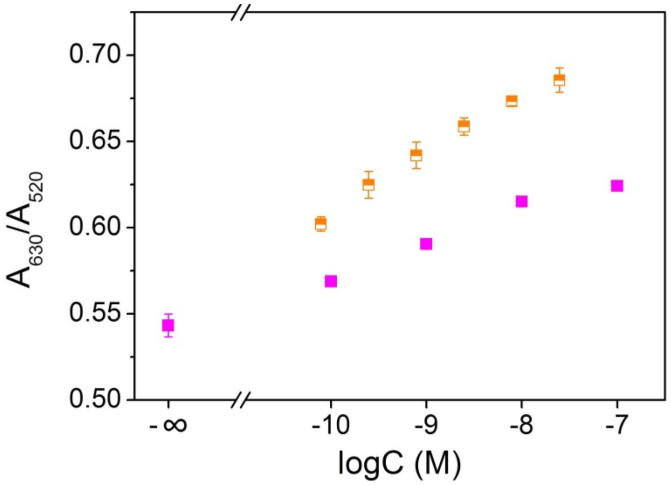

Figure S2: The A_{630}/A_{520} values of OTA (orange) and complimentary single-stranded DNA of the OTA aptamer (pink) in the control experiment.
