# Supplementary material for: Gold Nanoparticle-Aptamer-Based LSPR Sensing of Ochratoxin A at a Widened Detection Range by Double Calibration Curve Method
Source: Front Chem. 2018 Apr 4;6:94. doi: 10.3389/fchem.2018.00094 (PMC5893832; doi:10.3389/fchem.2018.00094)
Supplement: Supplementary file 3 [file Image3.PDF]

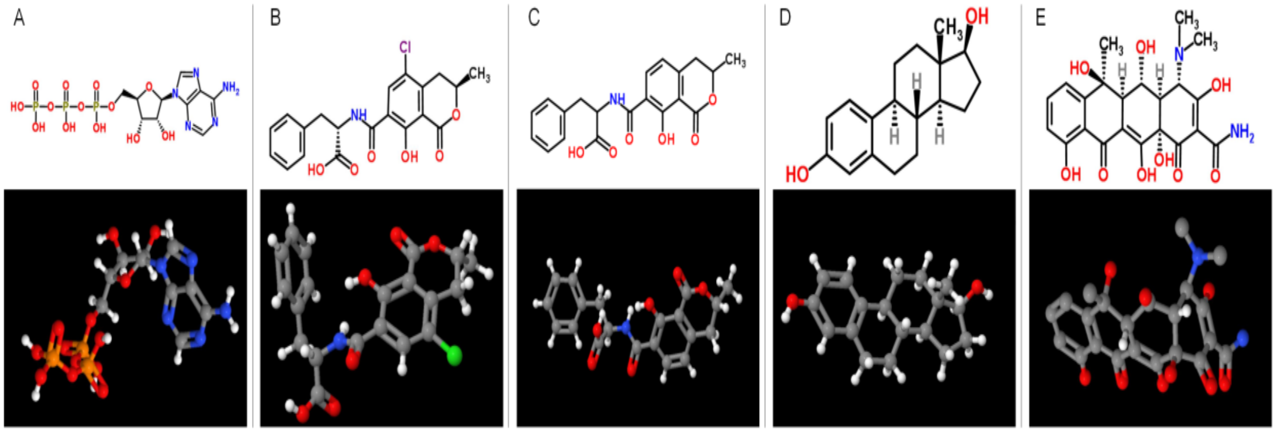

Figure S3: The structure of A: ATP; B: OTA; C: OTB; D: EST and E: OTC. Pictures are obtained from ChemSpider (www.chemspider.com).
