# Supplementary material for: Gold Nanoparticle-Aptamer-Based LSPR Sensing of Ochratoxin A at a Widened Detection Range by Double Calibration Curve Method
Source: Front Chem. 2018 Apr 4;6:94. doi: 10.3389/fchem.2018.00094 (PMC5893832; doi:10.3389/fchem.2018.00094)
Supplement: Supplementary file 4 [file Table1.DOCX]

**Table S1.** Nucleotide sequences of the prepared ATP, EST and OTC aptamers

| Name | Sequence (5ʹ to 3ʹ) | Reference |
| --- | --- | --- |
| ATP aptamer | ACCTGGGGGAGTATTGCGGAGGAAGGT | ([Huo et al., 2016](#_ENREF_2)) |
| EST aptamer | GCTTCCAGCTTATTGAATTACACGCAGAGGGTAGCGGCTCTGCGCATTCAATTGCTGCGCGCTGAAGCGCGGAAGC | ([Dong Huy et al., 2011](#_ENREF_1)) |
| OTC aptamer | CGTACGGAATTCGCTAGCGGGCGGGGGTGCTGGGGGAATGGAGTGCTGCGTGCTGCGGGGATCCGAGCTCCACGTG | ([Niazi et al., 2008](#_ENREF_3)) |

Dong Huy, G., Jin, N., Yin, B., and Ye, B. (2011). A novel separation and enrichment method of 17β-estradiol using aptamer-anchored microbeads. *Bioproc. Biosyst. Eng.* 34(2)**,** 189-195. doi: 10.1007/s00449-010-0460-4.

Huo, Y., Qi, L., Lv, X., Lai, T., Zhang, J., and Zhang, Z. (2016). A sensitive aptasensor for colorimetric detection of adenosine triphosphate based on the protective effect of ATP-aptamer complexes on unmodified gold nanoparticles. *Biosens. Bioelectron.* 78**,** 315-320. doi: 10.1016/j.bios.2015.11.043.

Niazi, J., Lee, S., Kim, Y., and Gu, M. (2008). ssDNA aptamers that selectively bind oxytetracycline. *Bioorgan. Med. Chem.* 16(3)**,** 1254-1261. doi: https://doi.org/10.1016/j.bmc.2007.10.073.
