# Supplementary material for: Gold Nanoparticle-Aptamer-Based LSPR Sensing of Ochratoxin A at a Widened Detection Range by Double Calibration Curve Method
Source: Front Chem. 2018 Apr 4;6:94. doi: 10.3389/fchem.2018.00094 (PMC5893832; doi:10.3389/fchem.2018.00094)
Supplement: Supplementary file 5 [file DataSheet1.DOCX]

# Steps of washing-off experiment

Step 1: add 500 μL OTA solution (10^-5^ g/mL) to 2 mL AuNP colloid, mix well and incubate for 10 min to get solution A; then add 15 μL NaCl solution (0.5 M) to 250 μL of solution A, incubate for 3 min and then measure the A_630_/A_520_ value.

Step 2: centrifuge the remaining solution A at 18000 rpm for 10 min and discard the supernatant, then resuspend the precipitate with ultrapure water to the volume before centrifugation to get solution B; after that, repeat the addition of NaCl, incubation, and measurement of A_630_/A_520_ value.

Step 3: repeat the centrifugation and resuspension to get solution C, and then repeat the addition of NaCl, incubation, and measurement of A_630_/A_520_ value.
